# Detection of *Rickettsia* spp. in Ticks from Domestic Dogs Living in Seven Major Indigenous Communities of Rio de Janeiro State, Brazil

**DOI:** 10.3390/pathogens15090991

**Published:** 2026-09-19

**Authors:** Amanda Scardini Leon Isaac, Tiago Marques dos Santos, Gabriel Batista da Silva Leão, Thaís Silva Oliveira, Maria Angélica da Silva Ferreira, Cheryl Gouveia, Thaina Aparecida Pereira Moura Cerqueira, Jhone Inácio Silveira, Leandro Meneguelli Biondo, Louise Bach Kmetiuk, Claudia Bezerra da Silva, Bruna de Azevedo Baêta, Carla de Freitas Campos, Alexander Welker Biondo, Matheus Dias Cordeiro

**Affiliations:** 1 Department of Epidemiology and Public Health, Federal Rural University of Rio de Janeiro (UFRRJ), Seropedica 23890-000, RJ, Brazil; amandascardini.inea@gmail.com (A.S.L.I.); tiagorural@gmail.com (T.M.d.S.); tholiveira.vet@gmail.com (T.S.O.); mariaasfcontato@gmail.com (M.A.d.S.F.); cheryl.gouveia@ufrrj.br (C.G.); mathcordeiro@hotmail.com (M.D.C.); 2 Department of Animal Parasitology, Federal Rural University of Rio de Janeiro (UFRRJ), Seropedica 23890-000, RJ, Brazil; gabrielbsleao.biologia@gmail.com (G.B.d.S.L.); cerqueirathaina23@gmail.com (T.A.P.M.C.); claudia_ufrrj@yahoo.com.br (C.B.d.S.); babaeta@hotmail.com (B.d.A.B.); 3 Special Indigenous Health District (SIHD)-South Coast, Brazilian Ministry of Health, Curitiba 80320-100, PR, Brazil; jhone.silveira@saude.gov.br; 4 Interdisciplinary Studies Graduate Program, College of Graduate Studies, University of British Columbia, Kelowna, BC V1V 1V7, Canada; leandromet@gmail.com; 5 Department of Veterinary Medicine, Federal University of Paraná (UFPR), Curitiba 80035-050, PR, Brazil; louisebachk@gmail.com; 6 Institute for Biomodels Science and Technology, Oswaldo Cruz Foundation (FIOCRUZ), Rio de Janeiro 21040-900, RJ, Brazil; carla.campos@fiocruz.br

**Keywords:** *Rickettsia* spp., ticks, indigenous communities, public health, Atlantic

## Abstract

(1) Background: Although Brazilian indigenous communities of Rio de Janeiro State, southeastern Brazil, have been mostly located in conservation units and more likely exposed to ticks and tick-borne diseases, such environmental exposure remains to be fully established. Accordingly, this study aimed to detect *Rickettsia* spp. in ticks collected from dogs in all indigenous communities of Rio de Janeiro State. (2) Methods: After taxonomic identification, the total DNA was submitted to PCR amplification for DNA fragment (ITS-2 gene), *gltA* gene and the *ompA* gene to *Rickettsia* spp. identification. (3) Results: The manuscript herein surveyed seven indigenous communities, had 171 dogs examined, 26 infested, yielding 70 ticks (predominantly *Amblyomma ovale*, 60/70 (85.7%), with *Rickettsia* detected in ticks from two communities and sequencing identifying *Rickettsia parkeri* strain Atlantic Rainforest. Molecular analyses revealed 19/70 (27.1%) *A. ovale* positive to *Rickettsia parkeri* strain Atlantic rainforest. (4) Conclusions: The present study has reported the circulation of *R. parkeri* strain Atlantic rainforest in indigenous communities of Rio de Janeiro. This study also reinforces the diverse range of ticks and *Rickettsia* species in indigenous community areas, underscoring the necessity for ongoing and regional surveillance.

## 1. Introduction

Obligate intracellular *Rickettsia* spp. are mainly transmitted by ticks and cause worldwide zoonosis [1,2]. Prior to 2009, *Rickettsia rickettsii*—the causative agent of Rocky Mountain spotted fever (RMSF) and Brazilian spotted fever (BSF)—was the sole tick-borne pathogenic *Rickettsia* recognized in Brazil. However, the epidemiological landscape shifted in 2010 when a novel spotted fever illness emerged in southeastern Brazil, subsequently designated as *Rickettsia* sp. strain Atlantic rainforest, reflecting the ecosystem where the initial transmission occurred [3].

In Brazil, Brazilian Spotted Fever is caused by *Rickettsia* species belonging to the Spotted Fever Group (SFG) and presents different clinical features, including a high case-fatality rate caused by *Rickettsia rickettsii*, and different clinical signs from mild to moderate non-lethal febrile disease and inoculation eschar caused by *Rickettsia parkeri* strain Atlantic rainforest [4].

Indigenous people have historically played a central role in maintaining ancestral and deeply integrated relationships with local ecosystems based on traditional practices of sustainable use [5]. Despite intense urbanization and habitat fragmentation, indigenous communities of Rio de Janeiro State, mostly of Guarani ethnicity, have preserved their way of life in close relation to the remaining native forests [6]. Such close and continuous contact between indigenous communities, natural environments, and wildlife may increase exposure to different vectors and pathogens [7].

A previous study reported *Rickettsia* spp. exposure in indigenous individuals, their dogs, and healthcare professionals from southern and southeastern Brazil, along with *Rickettsia* spp. infection in ticks collected from dogs [8]. In addition, other vector-borne pathogen exposures and infections have been reported in dogs from indigenous communities in central-western and northern Brazil [9,10], along with tick infestation. Although indigenous Brazilian communities in Rio de Janeiro State have historically been located in conservation units and are more likely to be exposed to ticks and tick-borne diseases, such environmental exposure remains to be fully established. Accordingly, the aim of this study was to assess the presence of *Rickettsia* spp. in ticks collected from dogs in the indigenous communities of Rio de Janeiro State, Brazil, and to analyze the associated eco-epidemiological characteristics of tick infection.

## 2. Materials and Methods

This research was conducted after approval by the Ethics Committee on Animal Use at Fiocruz (Protocol P-24/24.4).

Tick samples were collected from domestic dogs living in all seven major indigenous communities in Rio de Janeiro between February and August 2025 (Table 1).

The sampling strategy aimed to include at least 10% of the estimated dog population in each community to ensure representative coverage of the local canine population and to increase the likelihood of detecting circulating tick species and associated pathogens. Because no official data are available regarding dog population sizes in indigenous territories, the estimated number of dogs was calculated based on the human-to-dog ratio reported for Brazil, which was approximately 4:1 [8]. To adopt a conservative sampling framework, a 2:1 human-to-dog ratio was assumed for indigenous communities included in this study.

Dogs were manually restrained and inspected by certified veterinarians. Ticks were collected, placed in tubes containing a commercial stabilizer (RNAlater; Thermo Fisher Scientific, Waltham, MA, USA), and submitted for taxonomic identification [9]. After identification, ticks were frozen and kept at −20 °C in the same commercial stabilizer until molecular analysis. Dog owners responded to an epidemiological questionnaire on dog community access, forest entry habits, and hunting.

The ticks were washed with 1 mL of phosphate-buffered saline (PBS) and subjected to total DNA extraction using the phenol/chloroform method [10]. Tick DNA viability and PCR suitability were confirmed by amplifying a partial fragment of ITS-2 following an established protocol [11]. The samples were screened using primers CS-239 and CS-1069, which amplify an 834 bp fragment of the gltA gene (encoding the enzyme citrate synthase) specific to the genus *Rickettsia* [12]. The *omp*A gene of SFG was used as an additional target for confirmation and molecular characterization [13]. In summary, the amplification was performed under the following conditions: initial denaturation at 95 °C for 4 min, 40 cycles of 95 °C for 30 s, 52 °C for 30 s (gltA) and 55 °C for 30 s (ompA), 72 °C for 50 s, with a final extension at 72 °C for 5 min. The reactions were performed in a final volume of 25 μL with the reagents at the concentrations of 1U Taq polymerase (GoTaq DNA Polymerase; Promega, Madison, WI, USA), 0.8 µL of each primer, 0.2 mM of dNTP, 3 mM of MgCl2, 1X buffer (Colorless GoTaq, Promega^®^, Madison, WI, USA), 3 µL of test total DNA, and ultrapure water (q.s.) [14]. *Rickettsia vini* was used as the positive control, and ultrapure water was used as the negative control [15].

PCR products were applied to a 1.5% agarose gel, separated using electrophoresis, stained with ethidium bromide, and visualized using an ultraviolet light transilluminator. The PCR products were purified using a commercially available purifier (Exo-Sap-IT; GE Healthcare, Chicago, IL, USA). Fragments were sequenced in both directions using an automated genetic analyzer (ABI 3730 DNA Analyzer; Thermo Fisher Scientific) and Sanger sequencing was performed with the BigDye Terminator v3.1. The obtained sequences were aligned using the DNA Baser^®^ v5.11.3 program and subjected to a homology search with other sequences deposited in GenBank using the BLASTn tool. The datasets generated and analyzed in the current study were uploaded to the NCBI GenBank Nucleotide platform [https://www.ncbi.nlm.nih.gov/genbank/; accessed on 11 September 2026].

As ethical research counterpart and to ensure veterinary intervention, all dogs, participating or not in the herein survey, were offered and given an anti-rabies vaccination, dog species-specific vaccination, oral antiworm treatment, pour-on treatment for ticks and fleas, and dog treatment for sarcoptic mange and tungiasis, when necessary.

## 3. Results

**Figure 1:**
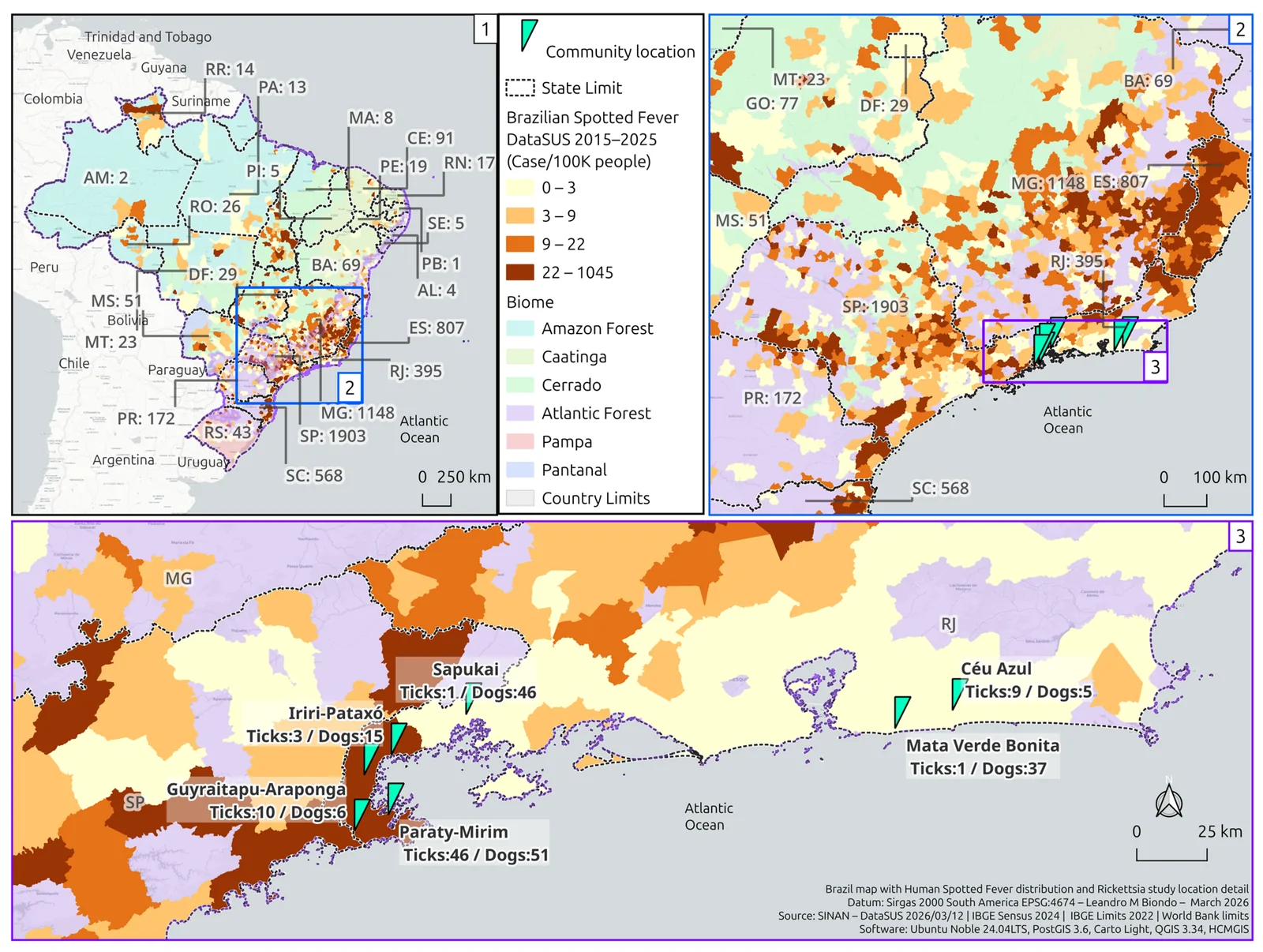
Location of the studied communities in Rio de Janeiro in the bottom map (**3**), which is part of the Atlantic Forest biome; labels indicate the number of ticks found and examined dogs. On the top left (**1**), the Brazilian territory shows its six land biomes overlaid with orange shades representing municipalities with Brazilian Spotted Fever cases per 100 thousand people (based on the 2024 Brazilian national census) from 2015 to 2025, closer details and the study location are shown on the top right (**2**). The State labels have the total cases in SINAN (DataSUS) database with geospatial information in the period.

In total, 171 domestic dogs belonging to 73 indigenous communities living in all seven indigenous communities of Rio de Janeiro were included in this study. Overall, 15.20% (26/171) were infested with ticks. A total of 70 ticks were collected, representing an average of 2.69 ticks per infested dog (minimum of one and maximum of six ticks per dog) (Table 2; Figure 1). All collected ixodid ticks were identified, with a total of 60 adult specimens of *Amblyomma ovale* (27 females and 33 males), one nymph of *Amblyomma sculptum*, and five nymphs and four adults (two females, two males) of *Rhipicephalus linnaei*. According to owners of examined dogs, only 15/171 (8.77%) lived inside households, 63/171 (36.84%) did not usually frequent the forest, and 98/171 (57.31%) did not have the habit of hunting.

The DNA of the identified ticks was tested, and the ITS-2 gene was successfully amplified. After analysis, 19/70 (27.14%) samples amplified the *gltA* gene of *Rickettsia* spp. and 10/19 (52.63% of *glt*A-positive samples) amplified the *omp*A gene. Observing only the species *A. ovale*, an amplification frequency of 31.67% (19/60) was obtained for *gltA* and 16.67% (10/60) for *ompA*. Only 2/7 communities have presented positive ticks for *Rickettsia* spp. (Paraty-Mirim and Araponga). Among them, six dogs tested positive for ticks, and five owners each presented with at least one animal (four males and two females). One female dog had 10 ticks (seven males and three females), and the other had two ticks testing positive for *glt*A in Paraty-Mirim and Araponga, respectively, whereas the others had only one tick per animal. The other three infected ticks were removed from the same female dog that presented with ten positive ticks during a subsequent visit.

Sequencing of the amplified products showed 100% identity with the gltA (781/781 bp) and ompA (499/499 bp) genes of *R. parkeri* strain Atlantic rainforest (CP040325). The datasets generated were successfully uploaded to the NCBI GenBank Nucleotide platform [16], which may be accessed through the corresponding accession numbers, including PZ067625-PZ067643 for *glt*A (19 identical sequences) and PZ067644-PZ067652 for *omp*A (nine identical sequences).

Three indigenous communities exhibited a homogeneous, preserved landscape, almost entirely covered by Atlantic Rainforest vegetation (Supplementary Figure S1). All seven locations are near native forest remnants and coastal areas subject to land-use restrictions designed to protect flora and fauna. These dense vegetation patches create a warm, humid microclimate that may favor tick occurrences among wildlife hosts, and domestic animals roaming into adjacent forest patches may carry ticks to humans. Céu Azul, Rio Pe-queno, Guyraitapú-Araponga and Mata Verde Bonita communities are situated in areas characterized predominantly by well-preserved forest environments and minimal or no agricultural activity. Paraty-Mirim and Sapukai are situated in transitional landscapes composed of forest fragments interspersed with agricultural areas and Iriri-Pataxó community represents an ecological transition area between the rainforest and coastal ecosystems.

## 4. Discussion

The variation in the degree of tick infestation in dogs in the seven indigenous communities can be explained by social and environmental factors. The study did not analyze the social factors that may explain the varying degrees of tick infestation in dogs in the seven indigenous communities. However, environmental characteristics and dog behavior can influence the dynamics of these tick infestations. In all communities, it was common for dogs to roam freely, at least as far as the forest edge. This behavior, combined with host diversity, may facilitate *A. ovale* [17]. Another factor was the prior administration of antiparasitic drugs to dogs in the Sapukai and Mata Verde Bonita communities by the municipalities responsible for the low levels of tick infestation.

This study represents the first molecular report of *R. parkeri* strain Atlantic rainforest in dog ticks from indigenous communities in Brazil, indicating circulation in such vulnerable populations.

Rickettsial surveys of dog ticks from indigenous communities should be particularly relevant, as dogs may play a role as “ecological bridges” of native rainforests and domestic household environments, even as sentinels of human exposure to infected ticks [18]. The Atlantic Rainforest areas have reported that most of *R. parkeri* strain Atlantic rainforest circulation is frequently associated with *A. ovale*, reinforcing the importance of eco-epidemiological surveillance in native forest environments [19,20].

All samples that tested positive for *glt*A, including those that tested negative for *omp*A, were sequenced successfully. The absence of *omp*A amplification in some samples may not rule out the presence of the spotted fever group, *Rickettsia*, as this gene is known to be more variable and less sensitive to PCR assays than the more conserved *glt*A gene. This discrepancy has been reported previously and is often attributed to differences in primer affinity, fragment size, and gene conservation [21]. Therefore, the *glt*A-positive/*omp*A-negative profile observed in this study was consistent with the methodological limitations rather than the absence of infection. Although mixed infections cannot be completely excluded using the conventional PCR approach, the sequencing quality obtained herein provides strong evidence that the analyzed samples were solely infected by *R. parkeri* strain Atlantic rainforest genotype.

As conventional PCR followed by direct sequencing may have limitations in detecting coinfections, additional *Rickettsia* species could be a possibility. However, co-infections involving distinct *Rickettsia* species typically generate overlapping peaks in Sanger sequencing chromatograms, with difficulty in sequence readability and no generation of a clear consensus sequence. Thus, coinfection was ruled out due to high-quality sequences yielded by all 19 *gltA*-and nine *OmpA*-positive amplicons, obtaining consensus sequences solely and consistently identified as *R. parkeri* strain Atlantic rainforest.

In the present study, all the chromatograms showed clean, well-defined, and unambiguous peaks, allowing the assembly of high-quality consensus sequences for the analyzed molecular markers. Therefore, the sequencing results strongly supported the interpretation that the obtained sequences corresponded to a single *Rickettsia* genotype.

We have previously reported *Rickettsia* spp. positivity in ticks from indigenous communities in southern and southeastern Brazil [8]. A total of 603 ticks were collected from dogs in indigenous communities, and 9/190 (4.7%) tested positive for fragment of *Rickettsia gltA* gene using qPCR [8]. This study has also described *Rickettsia* spp. seropositivity in 66/771 (8.6%) indigenous individuals, 9/99 (9.1%) healthcare professionals, and 116/386 (30.1%) dogs [8]. Combined with previous findings, our results reveal a diverse range of ticks but a single *Rickettsia* species within Brazilian indigenous communities in Rio de Janeiro, underscoring the necessity for ongoing regional surveillance.

Forest fragmentation can also influence *Rickettsia* spp. circulation, as larger and more connected forest fragments have been associated with a lower seroprevalence of *R. rickettsii* in dogs [22]. The presence of *A. ovale* ticks has been directly associated with the occurrence of carnivores (in the adult stage) and wild rodents (in the immature stage) in forest vegetation environments and found in low-altitude (maximum of 700 m) regions [23,24]. The study region contains a mountainous area of the Atlantic Forest below an altitude of 460 m. Forest fragmentation can also influence *Rickettsia* spp. occurrence, as larger and more connected forest fragments have been associated with a lower seroprevalence of *R. rickettsii* in dogs. Although this pattern has been described for *Amblyomma aureolatum* (found in high-altitude regions above 700 m), a tick with distinct ecological characteristics and similar epidemiological implications may be considered for *A. ovale*, given that both species are associated with wild carnivores and can bridge sylvatic and anthropogenic environments, thereby highlighting the role of forest–urban interfaces in *Rickettsia* spp. dynamics [25]. Such vulnerability has also been observed in indigenous communities, mostly those living in rudimentary conditions and immersed in an eco-epidemiological context that may increase direct tick exposure when entering forested areas or when tick-infested dogs return to their households [8].

All communities were located in the Atlantic Forest biome. The Céu Azul, Rio Pequeno, Guyraitapú-Araponga, and Mata Verde Bonita communities are situated in areas predominantly characterized by well-preserved forest environments and minimal or no agricultural activity. Paraty-Mirim and Sapukai are situated in transitional landscapes composed of forest fragments interspersed with agricultural areas and Iriri-Pataxó community represents an ecological transition area between the rainforest and coastal ecosystems. The detection of *Rickettsia* spp. only in tick-infested dogs does not allow us to establish a specific relationship between land-use patterns and the circulation of tick-borne rickettsiae.

One limitation is that the low adherence of indigenous communities to participation significantly compromised the representativeness of the study. Even with free-of-charge veterinary services, a lack of participation has already been reported in other indigenous communities and is attributed mostly to miscommunication, social isolation, and ethnic and cultural differences [26]. Another limitation is that most samplings performed in this study occurred in the high summer, with likely more tick infestations, dog examinations, and tick collections performed in a single incursion because of limitations and difficulties in organizing the taskforce to assist dog examinations and samplings. As all dogs (participating or not) in the present study were given anti-tick and anti-flea pour-on treatment (fipronil) after obtaining their owners’ consent, no additional tick samplings were conducted.

In addition, sampling only ticks infesting domestic dogs and testing them for *Rickettsia* spp. does not adequately reflect the relationship between land-use patterns and the risk of rickettsial diseases. Indigenous livelihood practices (forest foraging, hunting, and subsistence agriculture) affect tick spillovers to households.

## 5. Conclusions

In conclusion, this study represents the first molecular report of *R. parkeri* strain Atlantic rainforest in dog ticks from indigenous communities in Brazil, indicating circulation in such vulnerable populations. Future tick surveys in indigenous communities should be conducted in different seasons, including dogs and other (domestic, livestock, and wildlife) animal species, and other tick-borne pathogens should be surveyed as potential dog (and human) coinfections.

## Figures and Tables

**Table 1:** Summary of the seven major indigenous communities located in the Rio de Janeiro State.

Municipality *	Geolocation	Community	Predominant Ethnicity	Human Population	Estimate Dog Population (2:1)	Examined Dogs
Maricá 151 km (94 miles)	22°89′19.87′′ S 42°69′05.20′′ W	Céu Azul	Guarani Mbya	38	19	5
	22°57′17.25′′ S 42°53′26.96′′ W	Mata Verde Bonita	Guarani Mbya	133	67	37
Angra dos Reis 277 km (172 miles)	22°54′22.86′′ S 44°23′10.75′′ W	Sapukai	Guarani Mbya	420	210	46
	23°15′20.21′′ S 44°39′26.95′′ W	Paraty-Mirim	Guarani Mbya	205	103	51
Paraty 370 (230 miles)	23° 7′0.93′′ S 44°44′28.79′′ W	Rio Pequeno	Guarani Nhandewa and Mbyá	27	14	11
	23° 2′48.83′′ S 44°38′47.99′′ W	Iriri-Pataxó	Hã Hã Hãe	51	26	15
	23°18′41.10′′ S 44°46′30.36′′ W	Guyraitapu-Araponga	Guarani Mbya	19	8	6
			Total	893	447	171

* Distance from the Rio de Janeiro city.

**Table 2:** Ticks collected in the seven major indigenous communities of Rio de Janeiro State, their respective species, and the total number of positive ticks collected per community.

Municipality *	Community	Examined Dogs	Infested Dogs	Ticks	Tick Species	*glt*A Positive	*omp*A Positive
Maricá	Céu Azul	5	5	9	*Rhipicephalus linnaei*	0	
	Mata Verde Bonita	37	1	1	*Amblyomma sculptum*	0	
Angra dos Reis	Sapukai	46	1	1	*Amblyomma ovale*	0	
	Paraty-Mirim	51	14	46	*Amblyomma ovale*	16	8
Paraty	Rio Pequeno	11	0	0	NA	0	
	Iriri-Pataxó	15	2	3	*Amblyomma ovale*	0	
	Guyraitapu-Araponga	6	3	10	*Amblyomma ovale*	3	2
		171	26	70		19	

* Distance from the Rio de Janeiro city.

## Data Availability

All relevant data are within the manuscript.

## Supplementary Materials

The following supporting information can be downloaded at https://www.mdpi.com/article/10.3390/pathogens15090991/s1, Supplementary Figure S1. Sampling locations: 1. Brazil with recognized Indigenous Lands. 2. Location of the studied communities in the southeastern region, with map biomas land usage classes. 3. Rio de Janeiro State distribution of the studied communities. 4, 5 and 6. Regional detail of land usage on the three areas around the communities. And the local land usage of the communities: 7. Guyraitapu-Araponga. 8. Paraty-Mirim 9. Rio Pequeno. 10. Irixi-Pataxó. 11.Sapukai. 12. Mata Verde Bonita. 13. Céu Azul. Source: Mapbiomas land usage Collection 10 year 2024, Funai indigenous lands geoserver, IBGE state limits and World Bank country limits.
