# Supplementary material for: Detection of Rickettsia spp. in Ticks from Domestic Dogs Living in Seven Major Indigenous Communities of Rio de Janeiro State, Brazil
Source: Pathogens. 2026 Sep 19;15(9):991. doi: 10.3390/pathogens15090991 (PMC13610777; doi:10.3390/pathogens15090991)
Supplement: Supplementary file 1 [file pathogens-15-00991-s001.zip › pathogens-4466928-supplementary.pdf]

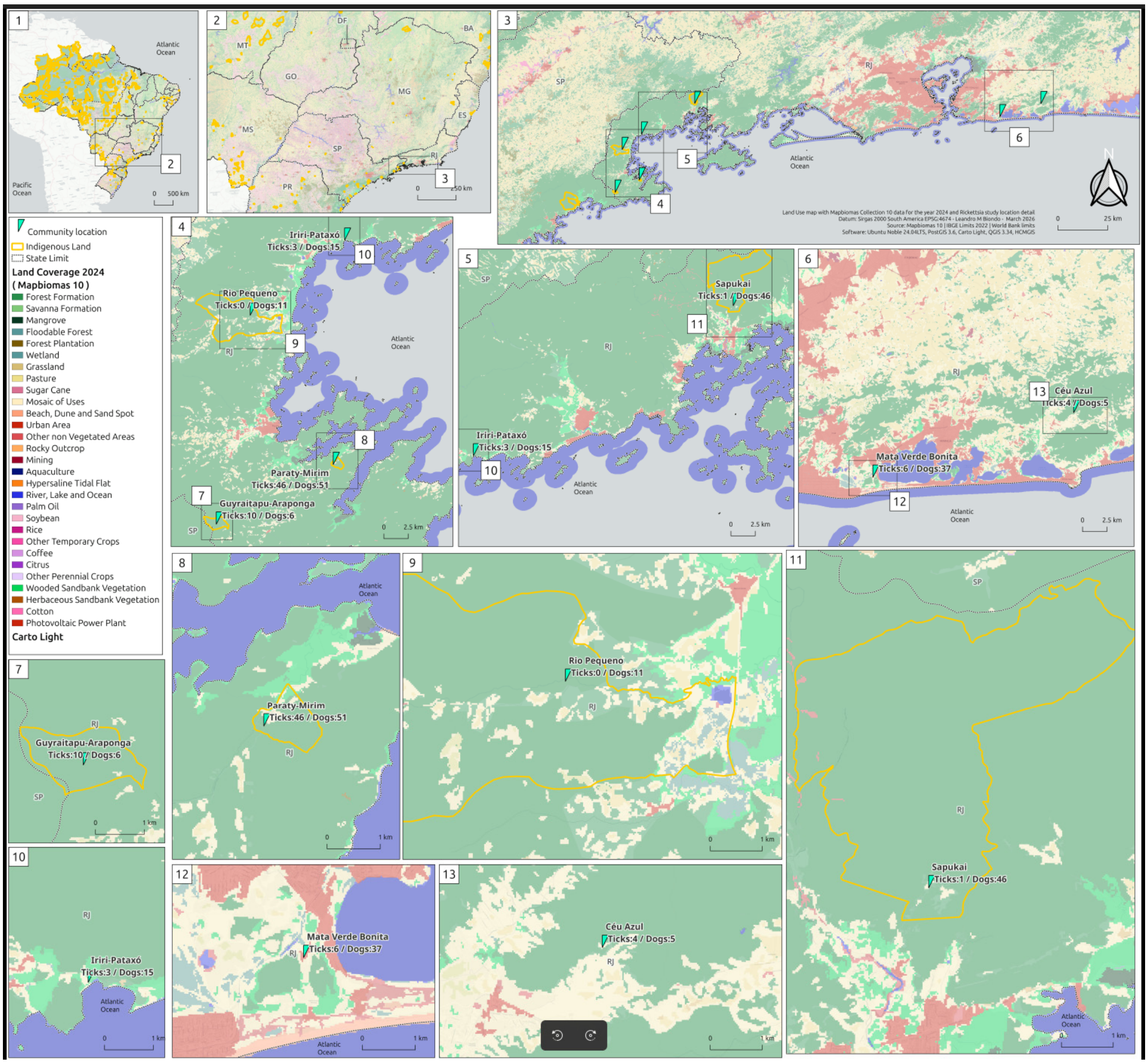

Figure S1. Sampling locations: 1. Brazil with recognized Indigenous Lands. 2. Location of the studied communities in the southeastern region, with map biomas land usage classes. 3. Rio de Janeiro State distribution of the studied communities. 4, 5 and 6. Regional detail of land usage on the three areas around the communities. And the local land usage of the communities: 7. Guyraitapu-Araponga . 8. Paraty-Mirim 9. Rio Pequeno. 10. Irixi-Pataxó. 11.Sapukai. 12. Mata Verde Bonita. 13. Céu Azul . Source: Mapbiomas land usage Collection 10 year 2024, Funai indigenous lands geoserver, IBGE state limits and World Bank country limits.
